# Exploring Polyurethane Elastomers as Flexible Punch Media for Microforming Applications: A Case Study of Extrusion–Cutting Process

**DOI:** 10.3390/mi17020230

**Published:** 2026-02-11

**Authors:** Chia-Ling Chen, Chuan-Hsiang Chang, Kuo-Ming Huang

**Affiliations:** 1 Department of Maritime Police, Central Police University, Taoyuan City 333322, Taiwan; chiaclloyd@gmail.com; 2 Department of Marine Engineering, National Taiwan Ocean University, No.2 Beining Road, Zhongzheng District, Keelung City 20224, Taiwan; boy8805183@gmail.com; 3 Research Center of Material Processing and Measurement, Department of Marine Engineering, National Taiwan Ocean University, No.2 Beining Road, Zhongzheng District, Keelung City 20224, Taiwan

**Keywords:** polyurethane elastomers, flexible punch media, microforming, extrusion–cutting process, material flow control

## Abstract

This study explores the feasibility of using polyurethane (PU) elastomer as a flexible punch-filling medium in cold forming. Microforming processes encounter challenges such as size effects and friction effects, which can lead to defects including fractures, distortions, and central depressions. The proposed method incorporates a high-hardness PU plate (95A) with excellent elasticity and near-incompressibility to address these issues. By compensating for axial compression through lateral expansion, the PU plate distributes pressure uniformly, reduces central stress, mitigates central acceleration effects, and minimizes defects caused by velocity gradients. Experiments and simulations using aluminum alloy Al 1050-O demonstrate that the PU-assisted extrusion–cutting process improves material flow, redistributes forming pressure, and enhances forming stability compared to conventional methods. This approach shows significant potential for advancing microforming technologies, particularly in industries requiring high-precision components.

## 1. Introduction

The advancement of miniaturization technologies has increased the strength requirements for mechanical components. However, microforming faces challenges such as size and friction effects, which significantly influence material flow and forming quality. Key factors include die–billet friction, grain size, surface roughness, and lubrication, with surface friction being the most critical. At the microscale, lubricants often fail, leading to material flow inconsistencies and defects such as fractures, distortions, and central depressions [1,2].

Advanced extrusion techniques have been developed to improve material microstructure and process efficiency. For example, Tesla’s adoption of a 6000-ton die-casting machine for vehicle body production has enhanced efficiency by integrating stamping and welding [3]. These large-scale manufacturing innovations provide insights for microforming applications, where similar principles of material flow control must be scaled down to microscopic dimensions. Despite these advancements, achieving uniform material flow is crucial, as undetected internal defects can compromise strength and cause fatigue failure. This challenge becomes more critical in microforming processes where size effects amplify the impact of material flow irregularities. Improper die design may result in metal flow stagnation and the formation of dead metal zones, leading to grain deformation and defects. Kim et al. [4] observed that forward extrusion, followed by backward extrusion, in automotive piston pin forming caused material stagnation in thin sections, resulting in defects. Such flow-related issues are particularly pronounced in microforming, where the increased surface-area-to-volume ratio makes uniform material distribution essential for maintaining component integrity at microscale dimensions.

Extrusion processes are extensively applied in industries such as automotive, medical, and aerospace to produce small, high-precision components. Key process parameters, including die design and material plasticity, are critical for achieving uniform strain distribution and preventing fractures. Kumar et al. [5] emphasized that controlling flow stress and strain rate is pivotal for achieving defect-free products in microforming.

**Figure 1:**
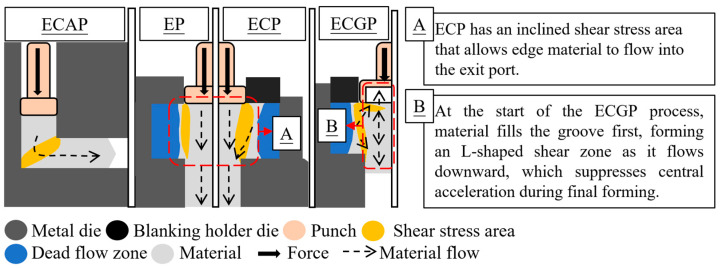
The illustration of various extrusion processes.

As illustrated in Figure 1, various extrusion technologies, including equal channel angular pressing (ECAP), extrusion process (EP), extrusion–cutting process (ECP), and the extrusion–cutting groove process (ECGP), have been developed to address these challenges. To enhance material flow and product quality, advanced negative-clearance die designs and ECP have been introduced. Specifically, ECP integrates negative clearance punches with hydrostatic pressure to suppress fractures and achieve a bright surface finish of up to 98%, surpassing traditional hydraulic blanking [6,7]. Similar to the ECAP process [8], the ECP has the potential to refine grains owing to the severe plastic deformation (SPD) induced during cutting and extrusion. This grain refinement enhances material strength and toughness, making it particularly advantageous for microforming.

The effectiveness of SPD techniques for grain refinement has been extensively validated. Horita [9] demonstrated that ECAP processing refined aluminum alloy grains to 500 nm through SPD-induced dynamic recrystallization. Dwiyati et al. confirmed that multi-pass ECAP processing of pure magnesium achieved ultrafine-grained structures, with grain refinement directly correlating to improved mechanical properties at the microscale [10]. These findings establish that ECP, employing similar SPD mechanisms, can achieve comparable grain refinement.

Recent advances in ECP have demonstrated its capability to produce ultrafine-grained structures through controlled SPD. Huang et al. [11] developed a movable container system integrating dual-stage stepped dies with PLC-controlled lateral compression to enhance material flow control. Progressive grain refinement was confirmed through EBSD analysis, demonstrating substantial hardness improvement compared to conventional cold extrusion. Additionally, Huang et al. [12] proposed the ECGP, which incorporates concave grooves at the punch end to address flow stagnation zones at the periphery, thereby accelerating central flow during punching. This design balances force distribution and reduces flow velocity disparities, improving overall forming quality.

Furthermore, Hsu et al. [13] developed a double trough die system for microforming, combining internal and external troughs to control material flow via hydrostatic pressure manipulation. The internal trough facilitates material compression and extrusion, whereas the external trough generates a hydrostatic pressure barrier that constrains lateral flow. This dual-trough configuration reduces the forming load by 15–20% compared with conventional flat dies and achieves dimensional accuracy within 5 μm (IT5–IT6 tolerance) in microgear manufacturing.

Despite these advancements, both the movable container system and the ECGP face critical limitations in microforming. The former requires complex PLC-controlled mechanisms and precise synchronization, which increases fabrication costs and reduces system reliability at the microscale. Similarly, the ECGP results in material waste due to the flow into concave grooves—a significant drawback where material utilization is critical. While each method aims to optimize material flow, ECP shows significant potential for further breakthroughs, provided that material flow and utilization efficiency are further improved.

## 2. Research Methods and Materials

### 2.1. Technical Principles

**Figure 2:**
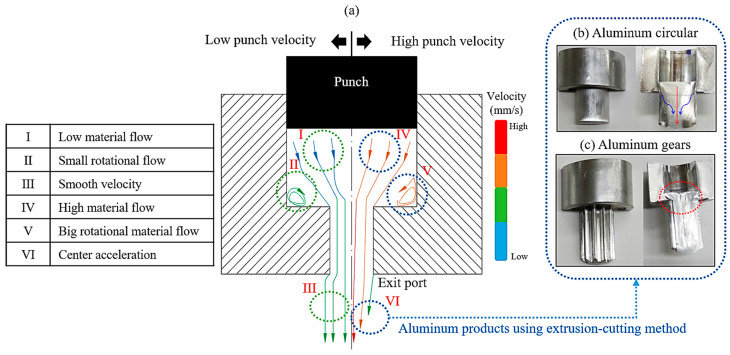
Comparison between flow conditions and product [12] (**a**) Correlation between material flow characteristics and punch velocity; (**b**) Aluminum circular product; (**c**) Aluminum gear product.

Conventional stamping processes typically involve multiple stages, including blanking, drawing, trimming, punching, and flanging, each of which requires dedicated dies and large mechanical presses. These processes incur high tooling and operational costs for individual products. Furthermore, unpolished or uncoated metal dies can create surface microvoids, leading to material flow instability, defects, and localized stress concentrations. To address these issues, die coatings are commonly applied to reduce friction and ensure smooth surfaces. However, repeated forming operations result in coating wear, necessitating periodic reapplication. The degree of wear is primarily influenced by the billet’s strength and the properties of the coating material [14,15]. To further reduce boundary friction, lubricants are frequently employed during forming operations. However, as product size decreases, lubrication effectiveness diminishes significantly, complicating material flow. Size effects alter surface stress distribution, while excess lubricant can accumulate within microvoids between the die and billet, forming “closed lubricant pockets” (CLP) that obstruct material flow and lead to defects. As shown in Figure 2, rapid extrusion creates a velocity gradient between the billet’s center and outer regions, with the center flowing faster than the outer regions [12]. This gradient can lead to defects, including central depressions, distortions, and fractures. Boundary friction, which intensifies when the billet is too small or when lubrication is insufficient, exacerbates these issues, collectively referred to as the friction effect [1,2].

To overcome the limitations of conventional friction-reduction approaches in microforming, alternative die design strategies have been explored to enhance material flow control and forming efficiency. Although the extrusion–cutting groove process (ECGP) and double-trough die systems have shown potential in mitigating central acceleration and controlling material flow through groove design, these configurations inherently entail partial material loss due to their specific groove geometries. Given that material utilization is critical to the economic and functional viability of microforming, such losses present a significant limitation. Concurrently, while movable container systems have demonstrated effectiveness in grain refinement through PLC-controlled lateral compression, their application in microforming remains constrained by challenges in achieving precise die miniaturization and the inherent complexity of equipment fabrication.

According to the Saint-Venant principle, external forces transmitted through contact surfaces significantly influence plastic flow behavior. Variations in external force conditions—such as those between rigid and flexible dies—affect material deformation. Flexible forming processes, categorized as contact-based or non-contact-based, adjust the spatial position and orientation of the forming tool, enabling precise control of localized plastic flow. However, increased flexibility often compromises the efficiency of forming. To address this, recent research has focused on integrating traditional rigid and flexible forming techniques or combining forming and non-forming processes to balance flexibility with efficiency [16].

Rubber-forming technology offers distinct advantages for the small-batch production of aerospace components. Sala [17] conducted a comprehensive numerical and experimental optimization of the Guerin process for aluminum alloy fuselage frames. By employing finite element analysis (FEA) integrated with Mooney–Rivlin hyperelastic constitutive models, the study demonstrated that rubber forming eliminates the need for precise punch–die alignment, thereby reducing tooling costs and enhancing surface finish compared to conventional stamping. Despite these benefits, inherent limitations remain, such as restricted forming pressures, limited drawing depth, and a propensity for wrinkling in deep-drawn regions subjected to compressive stresses. As a widely adopted flexible die method, rubber forming often functions as a compliant punch to ensure uniform pressure distribution. For instance, tube bulging techniques leverage the hyperelasticity of rubber to achieve isotropic pressure, facilitating the formation of complex geometries.

Zhang et al. [18] demonstrated that elastic rubber dies reduce maximum stress during forming compared to rigid metal dies while also minimizing springback. In aluminum plate bulging, using polyurethane (PU) rubber as a flexible forming medium significantly decreases plate thinning, minimizes material damage, and enhances forming capabilities compared to conventional hydraulic bulging techniques. Studies have shown that the friction coefficient and rubber hardness are critical parameters influencing forming results, with higher hardness rubbers effectively reducing plate thinning [19].

Polyurethane elastomers have emerged as promising flexible media for metal forming applications due to their unique mechanical properties and deformation characteristics. Prisacariu [20] documented the morphology-to-mechanical property relationships in PU elastomers, demonstrating that their segmented molecular structure provides an exceptional combination of elasticity, strength, and durability. These elastomers are characterized by their ability to undergo large elastic deformations while maintaining structural integrity, making them ideal for applications involving cyclic loading. The stress–strain behavior of thermoplastic polyurethanes, extensively studied by Qi et al. [21], exhibits highly nonlinear mechanical responses under both compression and tension. Their research revealed that PU elastomers possess an effective Poisson’s ratio approaching 0.5 (typically 0.49), signifying near-incompressibility under high compressive stress.

This characteristic enables PU materials to compensate for axial compression through lateral expansion, naturally flowing toward regions of lower pressure. Such a property is particularly advantageous for forming processes, as it ensures an isostatic-like pressure distribution across the contact surface, thereby mitigating localized stress concentrations that trigger defects. The selection of appropriate PU hardness is critical; according to Drobny [22], commercial-grade PU elastomers span a wide range of Shore hardness values (70A to 98A). Higher hardness grades (90A–95A) provide superior structural support and deformation resistance while maintaining sufficient elasticity for effective pressure redistribution.

Accordingly, this study evaluates PU plate-assisted ECP for microscale applications. Aluminum alloy Al 1050-O was selected for its high ductility and excellent formability, which are essential for electronic components and container manufacturing. Shore 95A PU plates serve as a flexible punch medium. Under the die constraint, the lateral expansion of the nearly incompressible polymer converts axial compression into enhanced hydrostatic pressure, ensuring uniform flow distribution and effectively suppressing the central flow acceleration effect.

**Figure 3:**
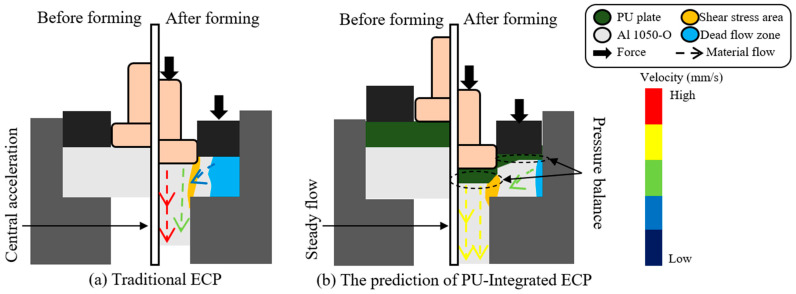
Schematic diagram of ECP.

As shown in Figure 3b, the PU plate is strategically positioned between the punch, blank holder, and billet to create a flexible and uniform pressure zone. The combined action of the blank holder force and the punch’s downward pressure forms a balanced pressure system, allowing the PU plate to evenly distribute the applied force across the material. This mechanism effectively reduces central stress, suppresses the central acceleration effect, and mitigates defects caused by velocity differences. By leveraging the unique properties of PU in flexible forming technologies, this approach enhances the precision and stability of microforming processes while addressing common defects.

### 2.2. Simulation Planning and Experimental Parameters

**Figure 4:**
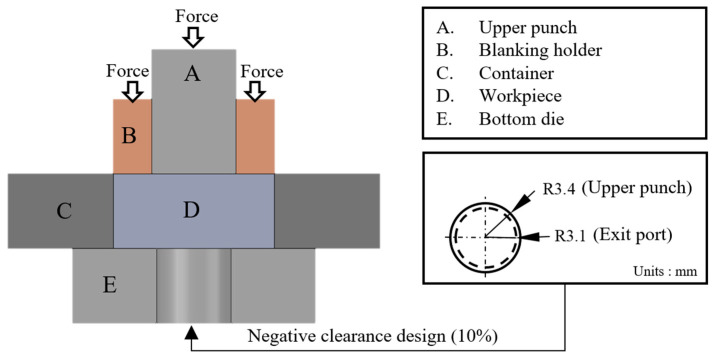
FEM simulation model.

Figure 4 illustrates the shearing extrusion forming process used in this study, which comprises a 10% negative clearance punch, a blank holder, and related components. The process involves applying a constant blanking holder force (details in Table 1) to stabilize the material, followed by the shearing extrusion operation. Negative clearance design reduces the gap between the punch and the material, lowering flow resistance and improving forming efficiency.

To analyze material flow and identify potential forming defects, numerical simulations were performed using DEFORM^®^ (Version V13.1.1), a finite element method (FEM) software package. The software was utilized to model the thermo-mechanical flow behavior, stress distribution, and deformation characteristics throughout the ECP, providing a theoretical foundation for the optimization of the forming parameters. The plastic deformation behavior of Al 1050-O was characterized using a power-law plasticity model, with material constants derived from established research [11,12,13].

(1) $$\sigma =K\cdot \varepsilon^{n}$$

where K = 130 MPa (strength coefficient), *n* = 0.26 (strain hardening exponent), and the yield stress was 20.2 MPa. In the numerical model, the billet was defined as a deformable plastic body, whereas all tooling components (dies and punches) were treated as rigid bodies to improve computational efficiency. Boundary conditions for the billet’s free surfaces were governed by contact constraints. Contact interactions were implemented using the penalty method, with interfacial friction characterized by a constant shear friction model:

(2) τ=m⋅k

where the interfacial friction factor (*m*) was set to 0.2395 for the metal dies. This value was determined through ring compression tests using R68 oil mixed with MoS_2_ as the lubricant.

Simulations were conducted at punch speeds of 0.1 mm/s, 0.5 mm/s, and 1.0 mm/s to evaluate material flow and central acceleration during the ECP without PU plates. These speeds were selected to represent a range of forming conditions: low-speed forming (0.1 mm/s) to minimize inertia effects and emphasize quasi-static material flow, high-speed forming (1.0 mm/s) to simulate dynamic conditions with pronounced central acceleration and stress concentration, and intermediate speed (0.5 mm/s) to observe the transition between these regimes. Experimental data, including extrusion length and forming force, were integrated into the simulations to refine parameters and enhance predictive accuracy.

PU plates, with their near-incompressibility under high compressive strain, play a key role in influencing material flow and reducing central acceleration. To isolate their effects, numerical simulations were first conducted exclusively for the baseline ECP without PU plates. Experimental investigations were then performed to evaluate the specific improvements provided by PU plate-assisted technology, particularly in mitigating central acceleration and enhancing forming quality. It should be noted that numerical simulations of the PU plates were not performed in this study, as the primary objective focused on the fundamental improvement of the EC process mechanics. The highly nonlinear behavior of PU elastomers under severe deformation, coupled with the complex fluid–structure interaction (FSI) at the workpiece interface, necessitates sophisticated constitutive modeling and entails prohibitive computational costs. Consequently, the efficacy of the PU plates was evaluated exclusively through experimental validation, providing a direct and practical demonstration of their performance in process optimization.

## 3. Simulation Results and Experimental Equipment

### 3.1. Simulation Results

**Figure 5:**
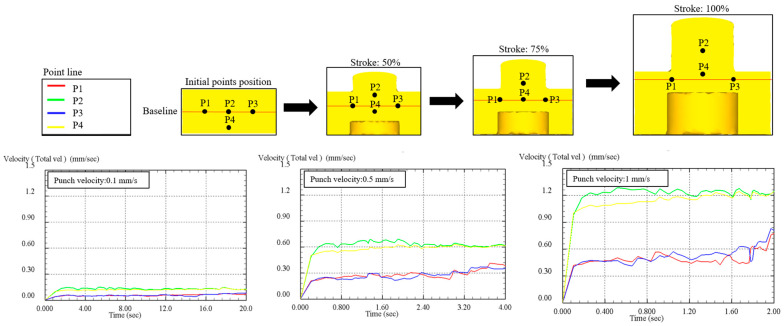
Evolution of the velocity distribution along the material’s flow path.

**Figure 6:**
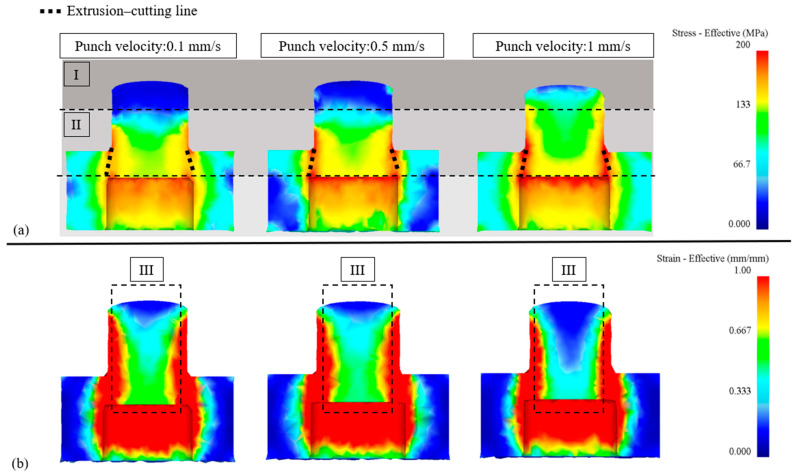
Shaded effective stress and strain distributions under different punch velocities. (**a**) Effective stress analysis; (**b**) Effective strain analysis.

The FEM simulations offered critical insights into the material flow behavior and stress distribution during the ECP. Analysis of varying punch speeds revealed significant stress concentration in the central region, particularly within the extrusion compression zone. This localized stress accumulation resulted in the formation of a “stress wall,” which obstructed the flow of surrounding material toward the outlet. The simulations demonstrated a clear relationship between punch speed and the development of central acceleration, providing a foundation for identifying potential defects and optimizing the forming process. The following sections separately address the material flow behavior and stress–strain distribution:

1. Material Flow Analysis: The relationship between central acceleration and forming speed was investigated using point-tracking methods, as shown in Figure 5. Using the line connecting points P1 and P3 as a reference, it was observed that at 50% of the stroke, point P2 had already moved significantly beyond the reference line, while point P4 approached the reference line at a relatively higher speed. At this stage, P2 had already exited through the outlet. By 75% of the stroke, P4 had nearly reached the reference line, and by the end of the forming process, P4 surpassed the reference line and exited the outlet. The point-tracking velocity curves further elucidate this behavior. During the initial stage of the forming process, both P2 and P4 exhibited a rapid velocity increase. Across all tested punch speeds, P2 consistently demonstrated a higher velocity than P4, emphasizing the phenomenon of central acceleration. This behavior indicates a material flow defect, where the outer material fails to flow smoothly into the central region. Consequently, this results in a persistent high-pressure concentration at the center, which can lead to defects in the forming process.

2. Effective stress/strain analysis: Figure 6a depicts the equivalent stress distribution at different punch velocities (0.1 mm/s, 0.5 mm/s, and 1 mm/s), revealing distinct variations between Regions I and II as the punch velocity increases. In Region I, higher punch velocities induce greater residual stresses in the extruded product due to significant compression of the central material. This is evident from the expansion of dark colored high-stress regions with increasing velocity. In Region II, stress concentration intensifies as punch velocity rises. At 0.1 mm/s, the extrusion cutting line primarily exhibits orange regions, indicating moderate stress levels and smoother material flow. At 1 mm/s, the cutting line becomes predominantly red, indicating elevated stress levels, while yellow stress regions penetrate deeper into the material, forming a “stress wall” that impedes material flow and causes stagnation. Figure 6b illustrates the equivalent strain distribution under the same conditions. In Region III, higher punch velocities lead to increased material accumulation near the outlet, as indicated by the prominent red and orange strain regions. After extrusion, the material flows outward along the edges, forming strain gradients that transition from red to orange and yellow. Strain accumulation near the outlet increases the risk of internal defects, such as microvoids and distortions, highlighting the critical influence of punch velocity on material flow behavior and defect formation.

3. Forming force analysis: Table 2 illustrates the forming forces at different punch speeds and varying stroke lengths. From the data, it can be observed that as the punch speed increases, the forming force also increases. Additionally, simulation results show that the extruded rod length increases with punch speed. This phenomenon supports the explanation of stress concentration, as higher punch speeds exacerbate the central acceleration effect, leading to greater localized stress, higher forming forces, and increased material extrusion.

In summary, the analysis of material flow, stress, and strain distributions at a punch velocity of 1 mm/s revealed critical issues, including stress concentration, strain accumulation, and defect formation near the extrusion–cutting line. Forming verification at this velocity was conducted to assess material behavior, providing a foundation for optimizing the process and improving defect reduction through enhancements to the PU material. To align with simulation results and avoid excessive stress that could intensify defect formation, 8000 N was selected as the maximum forming stopping force for the experiments. This value, derived from simulation analysis, was deemed sufficient to ensure effective material flow while mitigating stress concentration and strain accumulation. The use of 8000 N as the maximum force established a consistent and reliable baseline for evaluating the forming process and validating the effectiveness of PU material in improving forming quality.

### 3.2. Experimental Equipment and Parameters

The simulation results indicate that increasing punch speed accentuates central acceleration, leading to a significant rise in central stress and the formation of a “stress wall” near the shear zone. This obstructs material flow into the outlet, causing defects. To address this, the nearly incompressible property of the PU plate was utilized to uniformly distribute pressure, alleviate central stress, eliminate the stress wall, and improve material flow and forming quality. The PU plate dimensions (6.5 mm in diameter and 1.5 mm in thickness) were precisely engineered to match the billet geometry, ensuring full structural conformity with the billet’s top surface. During the compression phase, the near-incompressible nature of the PU elastomer triggers lateral expansion. When this expansion is restricted by the rigid die boundaries, the lateral confinement converts the deformation energy into an enhanced axial hydrostatic pressure. This mechanism significantly augments the forming force along the extrusion axis, thereby optimizing material flow control and ensuring a more uniform deformation profile.

**Figure 7:**
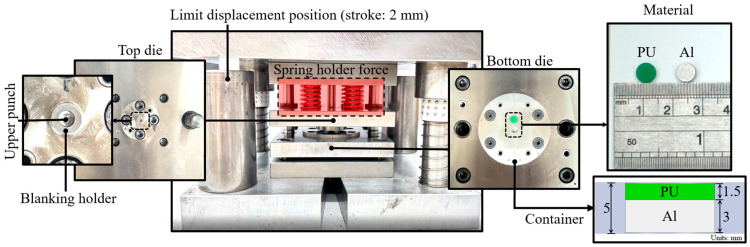
Experimental dies and materials.

Experiments were conducted using a GOTECH AI-7000-LAU servo-controlled testing machine (Gotech Testing Machines Inc., Taichung, Taiwan), applying a forming speed of 1 mm/s. A Sheng-Li spring provided a blanking holder force of 3400 N, and R68+ MoS2 lubricating oil minimized friction. To align with simulation results, the maximum forming force was set at 8000 N, ensuring effective material flow while reducing stress concentration and defects. Additionally, the die was equipped with a stopper to define the limit position of its displacement, with a stroke of 2 mm. The experimental die setup is shown in Figure 7. Final products were analyzed using a 2.5D image measuring instrument (Model EVM-S1510, RESSON Technology Co., Ltd., New Taipei City, Taiwan) to evaluate dimensional accuracy and forming quality. These experimental conditions, including the incorporation of the stopper to control the die’s displacement, ensured consistency with simulation results.

## 4. Experimental Results

### 4.1. Extrusion–Cutting Experiment

**Figure 8:**
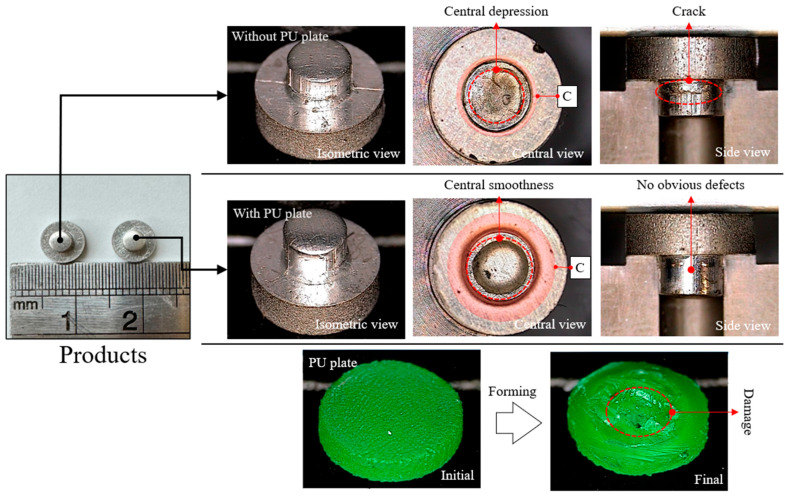
Mitigation of central defects and surface cracks using a PU plate in the ECP.

At a punch speed of 1 mm/s, the ECP exhibited significant stress concentration in the central region, primarily due to the central acceleration effect. This resulted in localized defects, including central depressions and surface cracks, on the formed product, as shown in Figure 8.

The maximum forming force was set at 8000 N, based on simulation results, which demonstrated that this force was sufficient to ensure effective material flow while minimizing stress concentration and defect formation. Experimental results confirmed that, at this speed, material flow was severely obstructed near the shear zone, forming a “stress wall” and resulting in defects. To address these issues, a PU plate was introduced. The nearly incompressible property of the PU plate enabled a more uniform redistribution of forming pressure, thereby reducing central stress and eliminating the “stress wall.” This uniform pressure enabled the material in contact with the blank holder’s surface to flow downward, slightly deforming the material into an inclined surface, as shown in the red-marked region at point C in Figure 8.

In contrast, in the absence of the PU plate, material flow was confined to the vicinity of the punch, characterized by localized deformation as the punch initially displaced the material downward. However, it was observed that the PU plate exhibited structural damage and plastic deformation in its central region. This was primarily attributed to the excessively tight extrusion–cutting clearance during punch compression. This narrow gap between the punch and the die induced mechanical interference and localized stress concentration at the interface, leading to subsequent wear and deformation of the PU medium. Despite this limitation, the implementation of the PU plate markedly enhanced the forming quality; it effectively mitigated central depressions, yielded a superior surface finish in the central region, and eliminated surface cracking, as corroborated by the experimental evidence in Figure 8.

This study emphasizes the importance of incorporating PU material into the ECP to optimize material flow, minimize defect formation, and achieve high-quality forming outcomes while also highlighting the need for optimized design and processing parameters to enhance PU plate durability and accommodate the demands of high-precision microforming operations.

### 4.2. Analysis of Experimental Forming Force Curves

**Figure 9:**
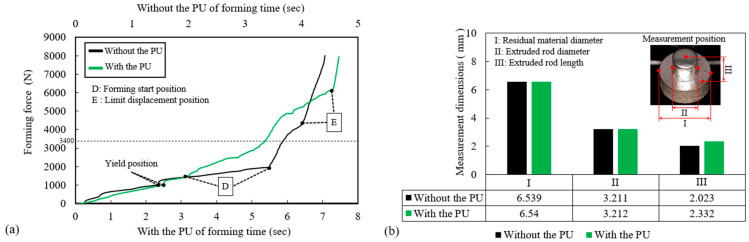
Comparative analysis of forming characteristics with and without PU plate; (**a**) Force-displacement relationships during forming process; (**b**) Dimensional accuracy of extruded components.

The presence of the PU plate increases the forming time compared to the process without it. This is attributed to the nearly incompressible nature of the PU material, which requires additional time for the forming force to generate and stabilize internal hydrostatic pressure. This pressure accumulation is essential for the uniform redistribution of forming stress, ensuring improved material flow and enhanced forming quality, as shown in Figure 9a. During the initial stage of forming, the forming force without the PU plate (black line) increases rapidly, while the force with the PU plate (green line) rises more gradually. At point D, which marks the start position of forming, the PU plate begins to accumulate hydrostatic pressure due to its nearly incompressible nature. This results in a smoother and more controlled increase in force during the early stage of forming. The PU plate facilitates a gradual buildup of hydrostatic pressure, which helps to uniformly distribute the forming stress and improve material flow. When the forming force exceeds the blanking holder force of 3400 N (dashed line), the punch initiates extrusion–cutting, beginning the material forming process. Due to the presence of the PU plate, the forming pressure is effectively distributed, resulting in a slower increase in force compared to the process without the PU plate. Additionally, the forming process with the PU plate reaches the die’s displacement limit position (point E) later than the process without the PU plate, indicating a more stable and controlled forming process.

To accurately evaluate forming performance, the dimensional features of the extruded specimens were characterized using a 2.5D vision measuring system (Model EVM-S1510, RESSON Technology Co., Ltd., New Taipei City, Taiwan). This system utilizes a CCD camera coupled with telecentric optics to capture specimen profiles with minimal optical distortion. Dimensional measurements were executed using high-precision edge detection algorithms; to ensure statistical reliability, each specimen was measured at three distinct locations to obtain an average value.

As illustrated in Figure 9b, a comparison of key dimensional features reveals distinct differences between the processes with and without the PU plate. While the residual material diameter (I) and the extruded rod diameter (II) remained nearly identical across both cases—measuring approximately 6.540 mm and 3.212 mm, respectively—the extruded rod length (III) exhibited a significant discrepancy. The PU-assisted process achieved a notably longer length of 2.332 mm, representing a 15.3% increase compared to the 2.023 mm obtained using the conventional rigid punch method. This enhancement underscores the superior material flow characteristics facilitated by the PU medium.

Furthermore, a discrepancy was initially observed between the simulated and experimental rod lengths, which was attributed to the deviation between the nominal and actual punch strokes. Since both processes were governed by a maximum forming force constraint of 8000 N, the actual punch stroke was limited by this force threshold rather than reaching the nominal target of 2.0 mm. Experimental data indicated that the actual extrusion depth was approximately 1.75 mm upon reaching the 8000 N limit. This deviation likely stems from machine compliance and the elastic deformation of the tooling system. By incorporating the measured stroke of 1.75 mm into the finite element model as a correction parameter, the revised simulation yielded a rod length of 2.09 mm. This result shows excellent agreement with the experimental value (2.15 mm), reducing the prediction error from 15.3% to 2.8%. These findings validate the numerical model’s predictive capability and confirm that the PU plate optimizes pressure distribution, leading to increased extrusion length and superior dimensional consistency under identical force conditions.

### 4.3. Experimental Validation and Performance Evaluation of PU-Assisted Forming

Experimental results obtained at a punch speed of 1.0 mm/s were compared with numerical simulations to validate the finite element model and identify potential defects under the prescribed process parameters. The close correspondence between experimental measurements and simulation predictions confirms the reliability of the numerical approach. Using this validated model as a baseline, the forming performance of the PU-assisted process was evaluated. To ensure a rigorous comparison, both the conventional rigid punch method and the PU-assisted process were executed under a consistent maximum forming force of 8000 N.

It is observed that the PU-assisted process requires a longer forming duration than the conventional method. This extended cycle is attributed to the incipient hydrostatic pressure establishment stage. At the onset of deformation, the PU plate undergoes compression, developing a hydrostatic pressure field that uniformly distributes the axial load across the workpiece surface. This uniform pressure effectively suppresses localized material flow acceleration. Unlike conventional rigid punch forming, where uneven flow leads to stress concentrations, the hyperelastic nature of the PU plate enables dynamic pressure redistribution. This self-regulating mechanism transfers excess pressure from high-stress zones to adjacent regions, significantly improving material flow uniformity and alleviating stress concentrations, thereby enhancing forming quality.

The results demonstrate that, under identical force constraints, the introduction of the PU plate markedly improves forming precision and increases the extruded length. This enhancement is rooted in the PU medium’s capability to homogenize material flow and stabilize the deformation process.

By employing ECP as a case study, this research establishes a proof-of-concept for integrating PU elastomers into extrusion-based microforming. The improvements in material flow uniformity, stress distribution, and surface integrity validate the technical viability of flexible punch media. Overall, this study constitutes a comprehensive feasibility assessment, providing a foundational basis for the industrial application of PU-assisted techniques in advanced precision manufacturing.

## 5. Conclusions and Future Work

### 5.1. Concluding Remarks

This study successfully demonstrates the feasibility and effectiveness of employing polyurethane (PU) elastomers as flexible punch media in microforming, specifically addressing critical challenges in the extrusion–cutting process (ECP) of aluminum alloy Al 1050-O. By integrating finite element method (FEM) simulations with experimental validation, this investigation provides a comprehensive understanding of PU-assisted forming mechanisms. The key findings are summarized as follows:

1. Material flow enhancement: The near-incompressible nature of PU elastomers (Poisson’s ratio ≈ 0.49) enables efficient pressure redistribution during deformation. Under axial compression, the lateral expansion of the PU plate—constrained by die boundaries—converts into a uniform axial pressure field. This mechanism reduces localized stress concentrations and promotes a more homogeneous strain distribution. By redistributing forming forces, the flexible medium effectively dampens velocity gradients between central and peripheral regions, thereby mitigating the central flow acceleration effect, which is a primary cause of defects in conventional rigid punch processes.
2. Defect mitigation: Comparative analysis reveals that PU-assisted forming effectively eliminates critical defects observed in conventional processes, such as central depressions, surface cracks, and stress-induced distortions. The flexible medium suppresses the formation of “stress concentrations” near the shear zone, facilitating a smoother material transition toward the extrusion outlet and ensuring superior surface integrity.
3. Dimensional performance: Under an identical maximum forming force of 8000 N, the PU-assisted process achieved a 15.3% increase in extruded rod length (2.332 mm vs. 2.023 mm) compared to the conventional rigid punch method, while maintaining comparable residual and extruded diameters. This improvement underscores the significantly enhanced material utilization efficiency.
4. Process stability and viability: Although the PU-assisted process requires a longer forming duration to establish hydrostatic pressure, this trade-off yields substantial benefits in forming precision and defect reduction—factors that are paramount in high-precision microforming.

The results validate PU elastomers as an innovative solution for overcoming size effects and friction-related challenges inherent in microscale manufacturing. This approach offers significant potential for advancing precision manufacturing in sectors requiring miniaturized components with stringent quality standards, such as the electronics, medical device, and aerospace industries.

### 5.2. Future Perspectives

While this study establishes the fundamental viability of polyurethane-assisted microforming, several research avenues merit further investigation for industrial implementation. Future work should focus on developing advanced PU formulations with enhanced tensile strength and fatigue resistance, optimizing die cavity geometries to minimize stress concentrations, and implementing hybrid punch architectures integrating PU flexibility with reinforced cores. Systematic evaluation across diverse workpiece materials (copper alloys, stainless steels, titanium alloys, and magnesium alloys) would broaden applicability. Exploring alternative flexible media with varying hardness grades (Shore 70A–98A) and chemical compositions (e.g., silicone elastomers, thermoplastic polyurethanes) could enable application-specific optimization. Design of Experiments (DOE) methodologies investigating interactions between forming force, punch velocity, PU plate thickness, die geometry, and temperature may provide valuable parameter optimization insights. Future phases could incorporate comprehensive FEM analysis by implementing sophisticated constitutive models (Mooney–Rivlin or Ogden). Through systematic investigation, PU-assisted microforming shows promise to transition from proof-of-concept to a robust manufacturing solution for precision component production across industrial sectors.

## Figures and Tables

**Table 1:** Parameters of the simulation and experiment.

Category	Parameter
Material	Al 1050-O
Working temperature	Cold working
Diameter of billet	6.5 mm
Thickness of billet	3 mm
Yield stress (y, MPa)	20.2
Strain hardening coefficient (n)	0.26
Strength coefficient (K, MPa)	130
Constant shear friction	0.2395
Blanking holder force	3400 N (fixed)
Negative clearance gap	10%
Upper punch of velocity	0.1, 0.5, 1.0 mm/s
Upper punch of stroke	2 mm
Flexible medium	Polyurethane 95A
Diameter of polyurethane	6.5 mm
Thickness of polyurethane	1.5 mm

**Table 2:** Forming loads at different punch velocities and stroke percentages.

	Punch Stroke	50%	75%	100%	
		Forming Force (N)			Extruded Length (mm)
Punch Velocity					
0.1 mm/s		5700	5800	7500	2.09
0.5 mm/s		6200	6200	7700	2.40
1.0 mm/s		6200	7000	8000	2.51

## Data Availability

The original contributions presented in this study are included in the article. Further inquiries can be directed to the corresponding author.

## Abbreviations

The following abbreviations are used in this manuscript:

PU	Polyurethane
ECAP	Equal channel angular pressing
EP	Extrusion process
ECP	Extrusion–cutting process
ECGP	Extrusion–cutting groove process
CLP	Closed lubricant pockets
FEM	Finite element method
Al 1050-O	Aluminum alloy 1050-O
95A	Shore hardness 95A
